# Nursing Practice of Airway Care Interventions and Prone Positioning in ICU Patients with COVID-19—A Dutch National Survey

**DOI:** 10.3390/jcm13071983

**Published:** 2024-03-29

**Authors:** Andrea A. Esmeijer, Fleur van der Ven, Eveline Koornstra, Laurien Kuipers, Paula van Oosten, Pien Swart, Christel M. Valk, Marcus J. Schultz, Frederique Paulus, Willemke Stilma

**Affiliations:** 1 Department of Intensive Care, Amsterdam University Medical Center, Location VUmc, 1081 HV Amsterdam, The Netherlandslaurienjanna@gmail.com (L.K.);; 2 Department of Intensive Care, Amsterdam University Medical Center, Location AMC, 1105 AZ Amsterdam, The Netherlands; 3 Department of Intensive Care, Rode Kruis Ziekenhuis, 1942 LE Beverwijk, The Netherlands; 4 Department of Intensive Care, Onze Lieve Vrouwe Gasthuis, Location ‘Oost’, 1091 AC Amsterdam, The Netherlands; e.j.koornstra@olvg.nl; 5 Mahidol-Oxford Research Unit (MORU), Faculty of Tropical Medicine, Mahidol University, Bangkok 10400, Thailand; 6 Nuffield Department of Medicine, University of Oxford, Oxford OX3 9DU, UK; 7 Division of Cardiac Thoracic Vascular Anesthesia and Intensive Care Medicine, Department of Anaesthesiology, General Intensive Care and Pain Medicine, Medical University Vienna, 1090 Vienna, Austria; 8 Center of Expertise Urban Vitality, Faculty of Health, Amsterdam University of Applied Sciences, 1105 BD Amsterdam, The Netherlands

**Keywords:** airway care interventions, prone positioning, invasively ventilated patients, COVID-19

## Abstract

**Background**: Airway care interventions and prone positioning are used in critically ill patients with coronavirus disease 2019 (COVID-19) admitted to the intensive care unit (ICU) to improve oxygenation and facilitate mucus removal. At the onset of the COVID-19 pandemic, the decision-making process regarding the practice of airway care interventions and prone positioning was challenging. **Objective:** To provide an overview of the practice of airway care interventions and prone positioning during the second wave of the pandemic in the Netherlands. **Method:** Web-based survey design. Seventy ICU nurses, each representing one intensive care in the Netherlands, were contacted for participation. Potential items were generated based on a literature search and formulated by a multidisciplinary team. Questions were pilot tested for face and construct validity by four intensive care nurses from four different hospitals. **Results:** The response rate was 53/77 (69%). This survey revealed widespread use of airway care interventions in the Netherlands in COVID-19 patients, despite questionable benefits. Additionally, prone positioning was used in invasively and non–invasively ventilated patients. **Conclusions:** The use of airway care interventions and prone positioning is time consuming and comes with the production of waste. Further research is needed to assess the effectiveness, workload, and environmental impact of airway care interventions and prone positioning.

## 1. Introduction

In critically ill patients with coronavirus disease 2019 (COVID-19) admitted to the intensive care unit (ICU) and requiring mechanical ventilation, airway care interventions and prone positioning are used to facilitate mucus removal and to improve oxygenation [1,2]. At the onset of the COVID-19 pandemic, ICU healthcare professionals faced an unprecedented situation due to a surge in patients with acute respiratory failure in need of mechanical ventilation. Uncertainty surrounded this novel disease, presenting intensivists and intensive care nurses with significant challenges [3,4]. The decision-making process regarding the practice of airway care interventions and prone positioning was challenging, given the novelty of the disease and the heightened contamination risks within the ICU environment [4,5,6].

Commonly used airway care interventions include endotracheal suctioning [7], humidification of the inhaled air [8,9], cough assist techniques [10,11], and nebulization therapy [12,13]. The evidence to support the use of airway care interventions in these patients is scarce, evidence-based guidelines are lacking, and there is variation in practice complicating treatment decisions [12,14,15].

The role of prone positioning, which involves turning patients to improve oxygenation [16,17], has gained prominence in the management of respiratory failure associated with COVID-19 [18,19]. Prone positioning improves oxygenation and enhances mucus mobilization [16,17]. This could potentially be explained by multiple aspects [20]. Firstly, it prevents atelectasis caused by the pressure of the heart on the dorsal lung tissue. Additionally, it improves chest wall compliance and ensures a better distribution of air, leading to a better ventilation−perfusion ratio. Finally, the downward direction of the trachea could enhance sputum mobilization. Prone positioning has important implications for the care process, impacting nursing care especially in the prevention of complications. Repositioning the patient involves ensuring proper alignment and support to prevent musculoskeletal injuries such as joint dislocation or nerve compression. Additionally, monitoring for complications such as pressure injuries, particularly to the face, is essential [21]. Careful positioning could enhance safety and patient comfort, potentially enabling the patient to maintain the position for a longer period of time [22]. In the Netherlands, the majority of invasively ventilated patients with COVID-19 were placed in prone position [23]. During the second wave of the pandemic, non-invasively ventilated patients were also placed in prone position while receiving oxygen therapies, like high flow nasal oxygen (HFNO) or non-rebreathing masks, so called ‘awake proning’ [23,24]. As such, understanding the practice of prone positioning alongside other airway care interventions is important for optimizing patient outcomes.

This survey aimed to provide an overview of the practice of airway care interventions and prone positioning in critically ill COVID-19 patients admitted to ICUs during the second wave in the Netherlands.

## 2. Materials and Methods

### 2.1. Study Design

We used a self-report web-based national survey design.

### 2.2. Survey Development and Formatting

The research team, consisting of physicians and registered nurses with extensive intensive care training and experience, iteratively developed the survey [25]. We generated potential items by searching for relevant studies in the MEDLINE and Cochrane databases during April and May 2021. We focused on airway care interventions and prone positioning in ICU patients with COVID-19. Questions centered on the following topics: (a) humidification, (b) endotracheal suctioning, (c) cough assist techniques, (d) nebulization therapy, and (e) prone positioning in invasively and non-invasively ventilated patients. The final survey contained 5 baseline questions and 23 questions related to the following topics: airway care interventions (10 questions), prone positioning (6 questions), and awake prone positioning (7 questions). Skip logic was used to enable the provision of questions based on participant responses to the preceding questions.

### 2.3. Survey Pilot Testing

The survey was pilot tested by 4 ICU nurses and 4 intensivists from 4 different hospitals. All pilot testers had experience in the care of COVID-19 patients and were working clinically. Every pilot tester completed a checklist on face and construct validity, including clarity, redundancy, completeness of items, and suggestions for additional items needed, as well as time to complete the survey. After pilot testing, minor revisions were made, and skip logic was corrected. The final survey questions are available through the corresponding author.

### 2.4. Sample

Our sample comprised all adult ICUs in the Netherlands. Each ICU was contacted by telephone to identify one ICU-nurse who would take responsibility for specific survey completion on behalf of their ICU. Respondents included preferably those responsible for nursing COVID-19 protocols and with clinical expertise in the care of COVID-19 patients.

### 2.5. Survey Administration

An instruction email with the secure link to the survey was sent to participants on 21 June 2021 and 2 completion reminders 10 and 12 days after, with the deadline being the 12 July 2021. The survey instructions explicitly stated the respondent was to report on the most recent practice of airway care interventions and prone positioning in COVID-19 patients, as described in ward protocol or treatment instructions on their ICU. All of the questions were labelled as mandatory. Participants were informed that their data would be processed anonymously.

### 2.6. Ethical Considerations

The Institutional Review Board of the Amsterdam University Medical Centers confirmed that the Medical Research involving Human Subjects Acts (WMO) did not apply, waiving the need for official approval (W21_327). Survey participation was voluntary, and consent was implied through the return of the survey. Neither patients nor public were involved in this survey.

### 2.7. Statistical Analysis

Frequencies and proportions were used to describe the categorical data. Proportions were reported as percentages. Practice of airway care interventions and prone positioning were reported per ICU. SPSS version 28 and R version 4.1.0 (R Core Team, Vienna, Austria) were used for the analysis of the results [26].

## 3. Results

### 3.1. Participants and Responses

Of all 77 contacted ICUs in the Netherlands, 53/77 (69%) provided survey responses. Individuals responding on behalf of their ICUs were most commonly ICU nurses (62%) and advanced ventilation nurse specialists (36%). Respondents represented both academic and non-academic hospitals. Details on the demographic characteristics of respondents are provided in Table 1.

### 3.2. Airway Care Interventions

Heated humidification was mentioned as being used routinely in most ICUs that participated in the survey. Nearly all ICUs used closed endotracheal suctioning systems. Just over half of the respondents reported not using manual hyperinflation in invasively ventilated patients with COVID-19. When used, this was mostly based on a clinical indication. Nebulization of bronchodilators was used in nearly all centers and 20.8% used concentrated saline. Details on the practice of airway care interventions are presented in Table 2.

### 3.3. Prone Positioning

Prone positioning of invasively ventilated patients was applied in all ICUs. The clinical indication to initiate prone positioning for these patients was mainly the PaO_2_/FiO_2_-ratio (P/F-ratio) (86.8%). The change in body position could be performed by a lateral turn towards a side every 3–4 h (32.1%) and could include repositioning of arms and head in so-called ‘swimming-positions’ every 4 h (60.4%). In some ICUs, the change of body positioning was less frequently applied. Multiple preventative measures for pressure ulcers were used, such as taping the eyes shut with ointment and eye pads (88.7%) and protection for cheeks and face (54.7%).

More than half of the respondents (52.8%) reported incorporating awake prone positioning in the care for non-invasively ventilated patients, although the estimated incidence was low. The primary clinical indicators to decide for awake prone positioning were P/F-ratio (43.4%), peripheral oxygen saturation (37.7%), and PaO_2_ (32.1%). HFNO (52.8%) was the predominant choice for oxygen therapy during awake prone position. The duration of awake proning was mostly guided by patients’ comfort (43.4%). There were no respondents reporting on protocolized preventative measures for pressure ulcers (35.8%) or specifically repositioning of the body (37.7%). Details on the practice of prone positioning are presented in Table 3.

## 4. Discussion

This is the first national survey on the practice of airway care and prone positioning for patients with COVID-19 during ICU stay in the Netherlands. It provides insight into nurse-driven practice regarding airway care and prone positioning after the initial wave during the COVID-19 pandemic. Invasively ventilated patients received predominantly heated humidification of inhaled air and closed systems for endotracheal suctioning. Additionally, more than half of the respondents reported the use of the relatively new treatment of awake prone positioning of non-invasively ventilated patients.

A notable proportion of respondents reported routine use of heated humidification, a trend consistent with findings in non-COVID-19 patients [15]. This preference could be driven by concerns that heat and moisture exchangers (HME) could potentially lead to an increase in dead space, although evidence did not confirm the relation between the dead space of HME and other respiratory variables [8,9]. Additionally, the challenge of managing sticky mucus in COVID-19 patients may have prompted the use of heated humidification [27]. However, its effect on rheology is unknown. While the rationale behind heated humidification is understandable, it is important to note that systematic reviews have demonstrated no superiority of heated humidification over HME in preventing complications or improving clinical outcomes [8,9]. Research indicates that the use of heated humidification can significantly escalate workload and costs [28]. Moreover, the adoption of heated humidification contributes to increased waste production and energy usage, underscoring the necessity to carefully weigh these factors for sustainable healthcare, especially considering the heightened demands and scarcities experienced during the pandemic.

The use of manual hyperinflation in this survey was primarily based on clinical indication or not applied at all. Our findings indicate a decline in manual hyperinflation use during the COVID-19 pandemic compared with previous surveys [15,29]. This decline may be attributed to increased awareness of the associated risks. Manual hyperinflation poses risks such as lung derecruitment due to loss of positive end-expiratory pressure, increased intrapulmonary pressures, and patient discomfort [10,30]. Furthermore, aside from the lack of evidence for its efficacy and its potential risks, an important consideration could have been the risk of contamination [10]. Manual hyperinflation involves disconnecting the tube system, which may expose healthcare workers to COVID-19 aerosols.

Another intervention, likely motivated by the desire to minimize the exposure of healthcare professionals to the potential risk of contamination with COVID-19 aerosols, is the reported use of closed suction systems by all respondents. It is noteworthy that in an earlier report closed suction systems failed to reduce cross-transmission in ICU patients [31]. Additionally, the American Association for Respiratory Care (AARC) 2022 guidelines state no significant differences in any outcome and recommends both systems to be safe and effective [32]. Despite the debate surrounding their efficacy, from an environmental perspective, the use of closed systems gains favor as a more sustainable choice, particularly in patients ventilated for longer durations [33], a scenario frequently encountered with COVID-19 patients.

The prevalence of sticky mucus in COVID-19 patients could be an explanation of nebulized medication we found in this survey, although it is comparable to reported practice before the pandemic [15,34]. However, it is crucial to note that despite this common practice, there is no evidence supporting the efficacy of routine use of nebulization in these cases. The decision to employ nebulization however should be approached with caution, as documented side effects such as agitation and cardiac arrhythmias have been reported [13]. Therefore, the nuanced consideration of potential benefits and risks in the context of nebulization practices is important.

Interestingly, one fifth of the ICUs reported using concentrated NaCl solutions as nebulization therapy, a practice that is relatively uncommon [34], and evidence regarding its effectiveness are lacking [35]. While there are no reported side effects of nebulized hypertonic saline [35], caution should be practiced as its safety and efficacy in critically ill patients have not been established. Until evidence supporting its safety and effectiveness in intubated patients emerges, we should refrain from using this therapy. Further research is needed to comprehensively assess its risks and benefits in this population. In line with international guidelines [18,19], this survey reports prone positioning was initiated in invasively ventilated patients with COVID-19. For patients in prone position, preventative measures for pressure ulcers varied per ICU. This variance could be explained by organizational factors and the availability of personnel. However, there also seems to be a lack of robust evaluations on the effect of repositioning frequency and positioning for pressure injury prevention and uncertainty about their effectiveness [36]. Regarding eye protection as part of care during prone positioning, a meta-analysis did not show an association between ocular injury in patients in relation to prone position, although ocular injuries during ICU stay are frequently not documented [37]. Nonetheless, these practices should not be neglected as pressure injuries are the most prevalent adverse events reported in pronated COVID-19 patients [38].

This survey sheds light on the relatively new therapy of awake prone positioning for non-invasively ventilated patients, revealing its utilization in nearly half of the surveyed ICUs. Circumstances like resource shortages and scarcity of ventilators are likely to have contributed to the increased adoption of this innovative intervention, as it offered a potential means to postpone or prevent respiratory insufficient patients from requiring intubation. Recent findings from systematic reviews and meta-analyses underscore the effectiveness of awake proning in improving oxygenation and reducing the need for endotracheal intubation [39,40]. The analyses did not show a clear advantage in terms of mortality or length of stay when compared with conventional care [39]. The initiation of awake proning requires careful patient assessment to identify those already in need of mechanical ventilation [41]. Determining the optimal timing, duration, and frequency of awake proning is crucial for organizing nursing care to ensure its safe and effective implementation [42]. It is essential to note that the introduction of awake proning necessitates additional monitoring and may come with an increased nurse workload [41].

### Strengths and Limitations

This survey has strengths. First, the input of experts in intensive care nursing and medicine contributed to the design of this survey. Additionally, the response rate of 69% was remarkable given the workload at the ICUs at that time. It could reflect a joint commitment to work together and improve ICU care. Finally, this survey received responses from a variety of Dutch ICUs and hospital types. This resulted in a generalizable comprehensive report on the practice of airway care interventions and prone positioning during the COVID-19 pandemic.

We acknowledge several limitations associated with the survey methodology used in this study. First, as is common with clinical practice surveys, there may be a discrepancy between the reported and actual clinical practice. The choice of self-report of practice inherently carries the risk of overestimation of one’s own practice, leading to potential self-reporting bias. Additionally, although we actively approached ICU professionals for participation, relying on a single individual’s report per ICU may have introduced reporting biases, as responses may reflect personal perceptions or a more favorable depiction of practices rather than accurately reflecting actual practices within that ICU. Second, this study focused exclusively on nursing practices related to airway care interventions and prone positioning, thereby excluding interventions performed by other healthcare professionals, such as physiotherapists. Furthermore, given the ongoing development of knowledge and practice in treating invasively ventilated patients with COVID-19, there is a possibility of time bias induced by the timing of the survey responses. Lastly, as this survey was exclusively distributed in the Netherlands, caution is warranted when generalizing our results to other countries.

## 5. Conclusions

Our survey indicates that in the Netherlands, the practice of airway care interventions in invasively ventilated COVID-19 patients included the prophylactic use of heated humidification and nebulization therapy, despite evidence of no benefit. Additionally, prone positioning was used in invasively and non–invasively ventilated patients. The use of airway care interventions and prone positioning is time consuming and comes with the production of waste. Further research is needed to assess the effectiveness of current nursing practices related to airway care and prone positioning. Additionally, attention should be paid to understanding the workload implications and environmental sustainability of these practices.

## Figures and Tables

**Table 1:** Demographic characteristics respondents and Dutch ICUs (*n* = 53).

Characteristics	*n* (%)
Respondent	
Advanced ventilation nurse specialist *	19 (36)
Advanced renal nurse specialist *	1 (2)
Advanced circulation nurse specialist *	-
Intensive Care nurse	33 (62)
Hospital type	
Academic hospital	5 (9)
Teaching hospital †	22 (42)
General hospital	26 (49)
Number of beds	
3–5	5 (9)
6–10	14 (26)
11–20	24 (45)
21–30	5 (9)
>30	5 (9)
Number of beds available for COVID-19	
3–5	-
6–10	9 (17)
11–20	18 (34)
21–30	13 (24.5)
>30	13 (24.5)

* ICU nurses with additional education 14-month program (240 study hours); † a non-academic hospital in which healthcare professionals are trained and educated; ICU, intensive care unit.

**Table 2:** Use of airway care interventions in COVID-19 patients in Dutch ICUs (*n* = 53).

Characteristics	*n* (%)
Airway humidification strategy	
Passively with heat and moisture exchanger (HME)	6 (11.3)
Actively with heated humidification	42 (79.2)
Actively on indication (e.g., purulent mucus or pulmonary oedema)	5 (9.4)
Endotracheal suction catheter	
Open system	1 (1.9)
Closed system	52 (98)
Manual hyperinflation	
Not applied	28 (52.8)
Yes, routinely (>1/day)	1 (1.9)
Yes, on indication only	24 (45.3)
Nebulization therapy	
No	2 (3.7)
Yes, routinely (>1/day)	14 (26.4)
Yes, on indication only	37 (69.8)
Use of nebulization therapy medication *	
Salbutamol	47 (88.7)
Salbutamol/Ipatropium (Combivent)	43 (81.1)
Ipatropium	50 (94.3)
Acetylcysteine (Fluimicil)	16 (30.2)
NaCl 3%	8 (15.1)
NaCl 5%	3 (5.7)

* multiple answers were possible.

**Table 3:** Use of prone positioning in invasively and non-invasively ventilated COVID-19 patients in Dutch ICUs (*n* = 53).

Characteristics	*n* (%)
Prone positioning	
Applied	53 (100)
Clinical indications used for decision to place patients in prone position *	
Peripheral oxygen saturation	26 (49.1)
ROX index	5 (9.4)
PaO_2_/FiO_2_-ratio (P/F-ratio)	46 (86.8)
SaO_2_/FiO_2_-ratio (P/F-ratio)	-
PaO_2_	26 (49.1)
FiO_2_	32 (60.4)
Dyspnea	7 (13.2)
Duration	
8 h	1 (1.9)
12 h	2 (3.8)
16 h	10 (18.9)
>16 h	40 (75.5)
Preventative repositioning measures pressure ulcers	
Change body position every 3–4 h	17 (32.1)
Change body position every 6–8 h	6 (11.3)
Change position arms and head every 4 h(‘swimming’)	32 (60.4)
Change position arms and head every 6 h(‘swimming’)	8 (15.1)
No use of protocol	7 (13.2)
Preventive measures pressure ulcers *	
Eyes taped shut with ointment and eye pads	47 (88.7)
Pressure ulcer protection cheeks and face	29 (54.7)
Pressure ulcer protection ears	21 (39.6)
Pressure ulcer protection knees	30 (56.6)
Use of skin cream/body lotion on skin	2 (3.8)
Use of barrier cream on skin	5 (9.4)
No use of protocol	-
Awake prone positioning	
Applied	28 (52.8)
Clinical indications used for decision to place patients in prone position *	
Peripheral oxygen saturation	20 (37.7)
ROX index	8 (15.1)
P/F ratio	23 (43.4)
S/F ratio	-
PaO_2_	17 (32.1)
FiO_2_	15 (28.3)
Dyspnea	10 (18.9)
Duration	
Minimum of 1 h	1 (1.9)
Minimum of 2 h	4 (7.5)
Minimum of 4 h	-
Based on patients’ comfort	23 (43.4)
Oxygen therapy interfaces **	
Nasal prong	4 (7.5)
Nasal cannula	2 (3.8)
Non-rebreathing mask	-
Nebulizer	-
High Flow Nasal Oxygen (HFNO)	28 (52.8)
Non-Invasive Ventilation (NIV)	1 (1.9)
Continuous Positive Airway Pressure (CPAP)	2 (3.8)
Preventative repositioning measures pressure ulcers *	
Change body position every 30 min	-
Change body position every hour	-
Change body position every 2 h	3 (5.7)
Patients change body position themselves	14 (26.4)
No use of protocol	19 (35.8)
Preventative measures pressure ulcers *	
Pressure ulcer protection cheeks and face	5 (13.9)
Pressure ulcer protection ears	3 (5.7)
Pressure ulcer protection knees	7 (13.2)
Use of skin crème/body lotion on skin	-
Use of barrier crème on skin	1 (1.9)
No use of protocol	20 (37.7)

* multiple answers were possible; ** respondents were requested to tick all options that apply; percentages may not total 100 because of rounding.

## Data Availability

The survey is in Dutch. Data are available upon request from the corresponding author.

## References

[1] Strickland S.L., Rubin B.K., Drescher G.S., Haas C.F., O’Malley C.A., Volsko T.A., Branson R.D., Hess D.R. (2013). AARC clinical practice guideline: Effectiveness of nonpharmacologic airway clearance therapies in hospitalized patients. Respir. Care.

[2] Fahy J.V., Dickey B.F. (2010). Airway mucus function and dysfunction. N. Engl. J. Med..

[3] Guan W.J., Ni Z.Y., Hu Y., Liang W.-H., Ou C.-Q., He J.-X., Liu L., Shan H., Lei C.-L., Hui D.S.C. (2020). Clinical Characteristics of Coronavirus Disease 2019 in China. N. Engl. J. Med..

[4] Wiersinga W.J., Prescott H.C. (2020). What Is COVID-19?. JAMA.

[5] Adhikari S.P., Meng S., Wu Y.-J., Mao Y.-P., Ye R.-X., Wang Q.-Z., Sun C., Sylvia S., Rozelle S., Raat H. (2020). Epidemiology, causes, clinical manifestation and diagnosis, prevention and control of coronavirus disease (COVID-19) during the early outbreak period: A scoping review. Infect. Dis. Poverty.

[6] Cummings M.J., Baldwin M.R., Abrams D., Jacobson S.D., Meyer B.J., Balough E.M., Aaron J.G., Claassen J., Rabbani L.E., Hastie J. (2020). Epidemiology, clinical course, and outcomes of critically ill adults with COVID-19 in New York City: A prospective cohort study. Lancet.

[7] Sole M.L., Bennett M., Ashworth S. (2015). Clinical Indicators for Endotracheal Suctioning in Adult Patients Receiving Mechanical Ventilation. Am. J. Crit. Care.

[8] Gillies D., Todd D.A., Foster J.P., Batuwitage B.T., Gillies D., Todd D.A., Foster J.P., Batuwitage B.T. (2017). Heat and moisture exchangers versus heated humidifiers for mechanically ventilated adults and children. Emergencias.

[9] Vargas M., Chiumello D., Sutherasan Y., Ball L., Esquinas A.M., Pelosi P., Servillo G. (2017). Heat and moisture exchangers (HMEs) and heated humidifiers (HHs) in adult critically ill patients: A systematic review, meta-analysis and meta-regression of randomized controlled trials. Crit. Care.

[10] Paulus F., Binnekade J.M., Vroom M.B., Schultz M.J. (2012). Benefits and risks of manual hyperinflation in intubated and mechanically ventilated intensive care unit patients: A systematic review. Crit. Care.

[11] Rose L., Adhikari N.K., Leasa D., Fergusson D.A., McKim D. (2017). Cough augmentation techniques for extubation or weaning critically ill patients from mechanical ventilation. Cochrane Database Syst. Rev..

[12] Ehrmann S., Roche-Campo F., Papa G.F.S., Brochard L.J., Apiou G. (2013). Aerosol therapy during mechanical ventilation: An international survey. Intensive Care Med..

[13] Van Meenen D.M., Van Der Hoeven S.M., Binnekade J.M., De Borgie C.A., Merkus M.P., Bosch F.H., Paulus F. (2018). Effect of On-Demand vs Routine Nebulization of Acetylcysteine With Salbutamol on Ventilator-Free Days in Intensive Care Unit Patients Receiving Invasive Ventilation: A Randomized Clinical Trial. JAMA.

[14] Ntoumenopoulos G., Hammond N., Watts N.R., Thompson K., Hanlon G., Paratz J.D., Thomas P. (2018). Secretion clearance strategies in Australian and New Zealand Intensive Care Units. Aust. Crit. Care.

[15] Stilma W., van der Hoeven S.M., Scholte Op Reimer W.J., Schultz M.J., Rose L., Paulus F. (2021). Airway Care Interventions for Invasively Ventilated Critically Ill Adults-A Dutch National Survey. J. Clin. Med..

[16] Baldi M., Sehgal I.S., Dhooria S., Agarwal R. (2017). Prone Positioning?. Remember ABCDEFG. Chest.

[17] Guérin C., Reignier J., Richard J.-C., Beuret P., Gacouin A., Boulain T., Mercier E., Badet M., Mercat A., Baudin O. (2013). Prone positioning in severe acute respiratory distress syndrome. N. Engl. J. Med..

[18] Alhazzani W., Evans L., Alshamsi F., Møller M.H., Ostermann M., Prescott H.C., Arabi Y.M., Loeb M., Gong M.N., Fan E. (2021). Surviving Sepsis Campaign Guidelines on the Management of Adults with Coronavirus Disease 2019 (COVID-19) in the ICU: First Update. Crit. Care Med..

[19] Panel C.-T.G. Coronavirus Disease 2019 (COVID-19) Treatment Guidelines. https://www.covid19treatmentguidelines.nih.gov/.

[20] Hadaya J., Benharash P. (2020). Prone Positioning for Acute Respiratory Distress Syndrome (ARDS). JAMA.

[21] Hughes P.J. (2023). Reducing Facial Hospital-Acquired Pressure Injuries Related to Prone Positioning in the Intensive Care Unit. J. Wound Ostomy Cont. Nurs..

[22] Oliveira V.M.D., Weschenfelder M.E., Deponti G., Condessa R., Loss S.H., Bairros P.M., Vieira S.R.R. (2016). Good practices for prone positioning at the bedside: Construction of a care protocol. Rev. Assoc. Méd. Bras..

[23] Stilma W., Van Meenen D.M., Valk C.M., De Bruin H., Paulus F., Serpa Neto A., Schultz M.J., PRoVENT-COVID Collaborative Group (2021). Incidence and Practice of Early Prone Positioning in Invasively Ventilated COVID-19 Patients-Insights from the PRoVENT-COVID Observational Study. J. Clin. Med..

[24] Bower G., He H. (2020). Protocol for awake prone positioning in COVID-19 patients: To do it earlier, easier, and longer. Crit. Care.

[25] Burns K.E., Duffett M., Kho M.E., Meade M.O., Adhikari N.K., Sinuff T., Cook D.J. (2008). A guide for the design and conduct of self-administered surveys of clinicians. CMAJ.

[26] (2021). R: A Language and Environment for Statistical Computing.

[27] Wang Y.B., Zhang M.B., Yu Y.B., Han T., Zhou J., Bi L. (2020). Sputum characteristics and airway clearance methods in patients with severe COVID-19. Medicine.

[28] Menegueti M.G., Auxiliadora-Martins M., Nunes A.A. (2016). Cost-Effectiveness Analysis of Heat and Moisture Exchangers in Mechanically Ventilated Critically Ill Patients. Anesthesiol. Pain. Med..

[29] Paulus F., Binnekade J.M., Middelhoek P., Schultz M.J., Vroom M.B. (2009). Manual hyperinflation of intubated and mechanically ventilated patients in Dutch intensive care units--a survey into current practice and knowledge. Intensive Crit. Care Nurs..

[30] Tucci M.R., Nakamura M.A., Carvalho N.C., Volpe M.S. (2019). Manual Hyperinflation: Is It Effective?. Respir. Care.

[31] Ramírez-Torres C.A., Rivera-Sanz F., Sufrate-Sorzano T., Pedraz-Marcos A., Santolalla-Arnedo I. (2023). Closed Endotracheal Suction Systems for COVID-19: Rapid Review. Interact. J. Med. Res..

[32] Blakeman T.C., Scott J.B., Yoder M.A., Capellari E., Strickland S.L. (2022). AARC Clinical Practice Guidelines: Artificial Airway Suctioning. Respir. Care.

[33] Stilma W., Esmeijer A., Paulus F., Frenzel T., Touw H., Stobernack T. (2023). Open Versus Closed Suctioning in Invasively Ventilated Critically Ill Patients for Sustainability of ICU Care: A Life-Cycle Assessment Comparison. Respir. Care.

[34] Ehrmann S., Roche-Campo F., Bodet-Contentin L., Razazi K., Dugernier J., Trenado-Alvarez J., Donzeau A., Vermeulen F., Thévoz D., Reva Research Network (2016). Aerosol therapy in intensive and intermediate care units: Prospective observation of 2808 critically ill patients. Intensive Care Med..

[35] Claudius C., Perner A., Moller M.H. (2015). Nebulised dornase alfa versus placebo or hypertonic saline in adult critically ill patients: A systematic review of randomised clinical trials with meta-analysis and trial sequential analysis. Syst. Rev..

[36] Gillespie B.M., Walker R.M., Latimer S.L., Thalib L., Whitty J.A., McInnes E., Lockwood I., Chaboyer W.P. (2021). Repositioning for pressure injury prevention in adults: An abridged Cochrane systematic review and meta-analysis. Int. J. Nurs. Stud..

[37] Patterson T.J., Currie P., Williams M., Shevlin C. (2021). Ocular Injury Associated With Prone Positioning in Adult Critical Care: A Systematic Review and Meta-Analysis. Am. J. Ophthalmol..

[38] Gabiatti D., Vieira L.G., Margatho A.S., dos Santos B.N., Clark A.M., Vasques C.I., de Campos Pereira Silveira R.C. (2024). Prevalence of adverse events in pronated intubated adult COVID-19 patients: A systematic review with meta-analysis. J. Clin. Nurs..

[39] Li Y., Zhao G., Ma Y., Wang L., Liu Y., Zhang H. (2024). Effectiveness and safety of awake prone positioning in COVID-19-related acute hypoxaemic respiratory failure: An overview of systematic reviews. BMC Pulm. Med..

[40] Tekantapeh S.T., Nader N.D., Ghojazadeh M., Fereidouni F., Soleimanpour H. (2024). Prone positioning effect on tracheal intubation rate, mortality and oxygenation parameters in awake non-intubated severe COVID-19-induced respiratory failure: A review of reviews. Eur. J. Med. Res..

[41] McNicholas B.A., Ibarra-Estrada M., Perez Y., Li J., Pavlov I., Kharat A., Vines D.L., Roca O., Cosgrave D., Guerin C. (2023). Awake prone positioning in acute hypoxaemic respiratory failure. Eur. Respir. Rev..

[42] Brockman A., Carmel R.L., Buchko B.L. (2022). Nurse-led implementation of awake prone positioning. Nursing.

